# Supplementary material for: NeST: nested hierarchical structure identification in spatial transcriptomic data
Source: Nat Commun. 2023 Oct 17;14:6554. doi: 10.1038/s41467-023-42343-x (PMC10582109; doi:10.1038/s41467-023-42343-x)
Supplement: Supplementary file 1 — Supplementary Information [file 41467_2023_42343_MOESM1_ESM.pdf]

**Supplemental Information**

# Nested Hierarchical Structure in Spatial Transcriptomic Data with NeST

Benjamin L. Walker[1,2], Qing Nie[1,2,3,*]

[1] The NSF-Simons Center for Multiscale Cell Fate Research, University of California Irvine, Irvine, CA, 92627, USA

[2] Department of Mathematics, University of California Irvine, Irvine, CA, 92627, USA

[3] Department of Developmental and Cell Biology, University of California Irvine, Irvine, CA, 92627, USA

[*] Corresponding Author: qnie@uci.edu

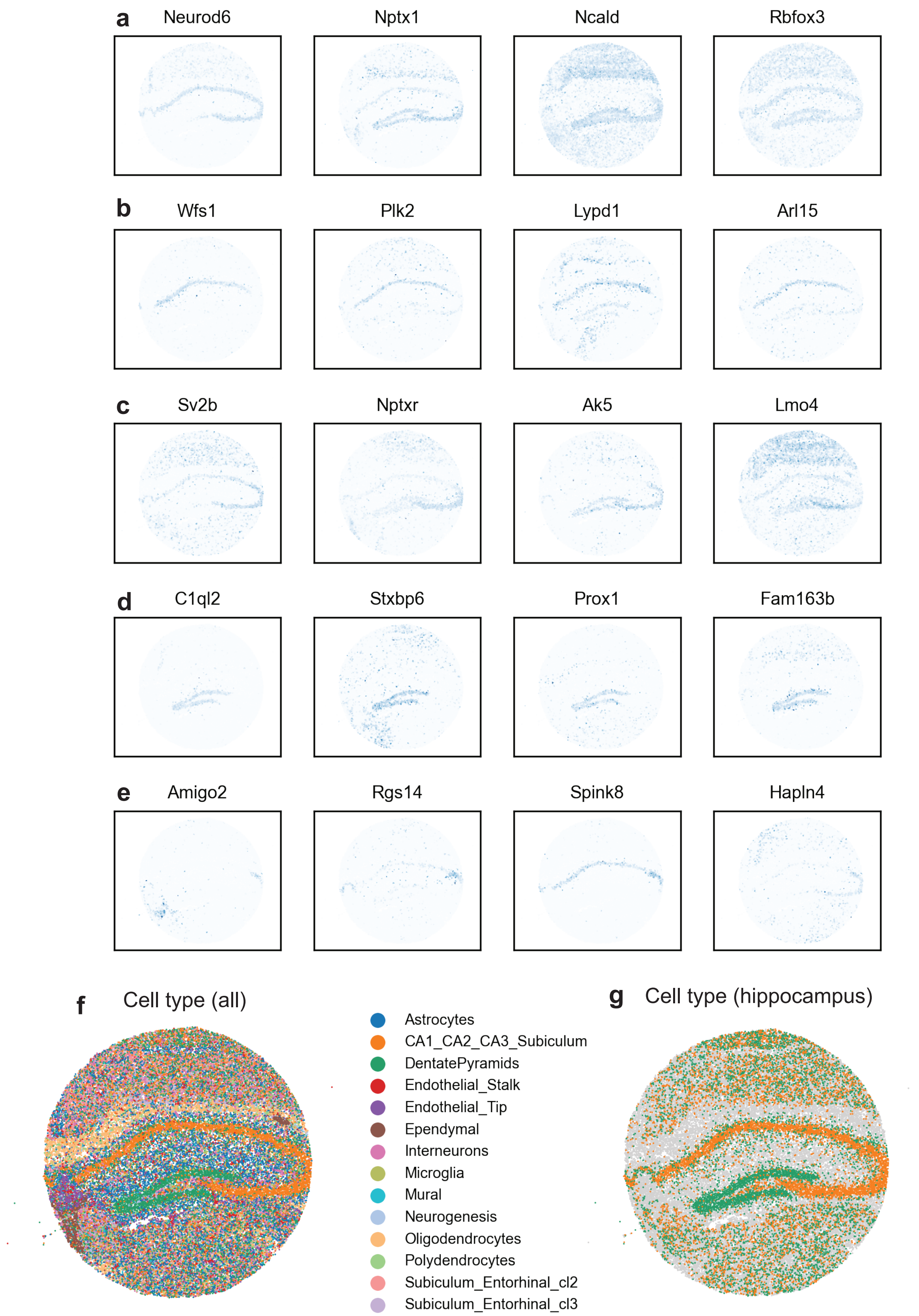
a
Neurod6
Nptx1
Ncald
Rbfox3
b
Wfs1
Plk2
Lypd1
Arl15
c
Sv2b
Nptxr
Ak5
Lmo4
d
C1ql2
Stxbp6
Prox1
Fam163b
e
Amigo2
Rgs14
Spink8
Hapln4
f
Cell type (all)
Astrocytes
CA1_CA2_CA3_Subiculum
DentatePyramids
Endothelial_Stalk
Endothelial_Tip
Ependymal
Interneurons
Microglia
Mural
Neurogenesis
Oligodendrocytes
Polydendrocytes
Subiculum_Entorhinal_cl2
Subiculum_Entorhinal_cl3
g
Cell type (hippocampus)

**Supplemental Figure 1:** Hierarchical marker genes for hippocampal structure, representing the hippocampus **(a)**, CA1 **(b)**, CA2 **(c)**, CA3 **(d)**, and dentate gyrus **(e)** regions. Color represents normalized gene expression. **f** Cell type annotation for all cells in dataset. **g** Highlighting only the cell types that should represent the hippocampus shows extensive presence of these cells elsewhere in the dataset.

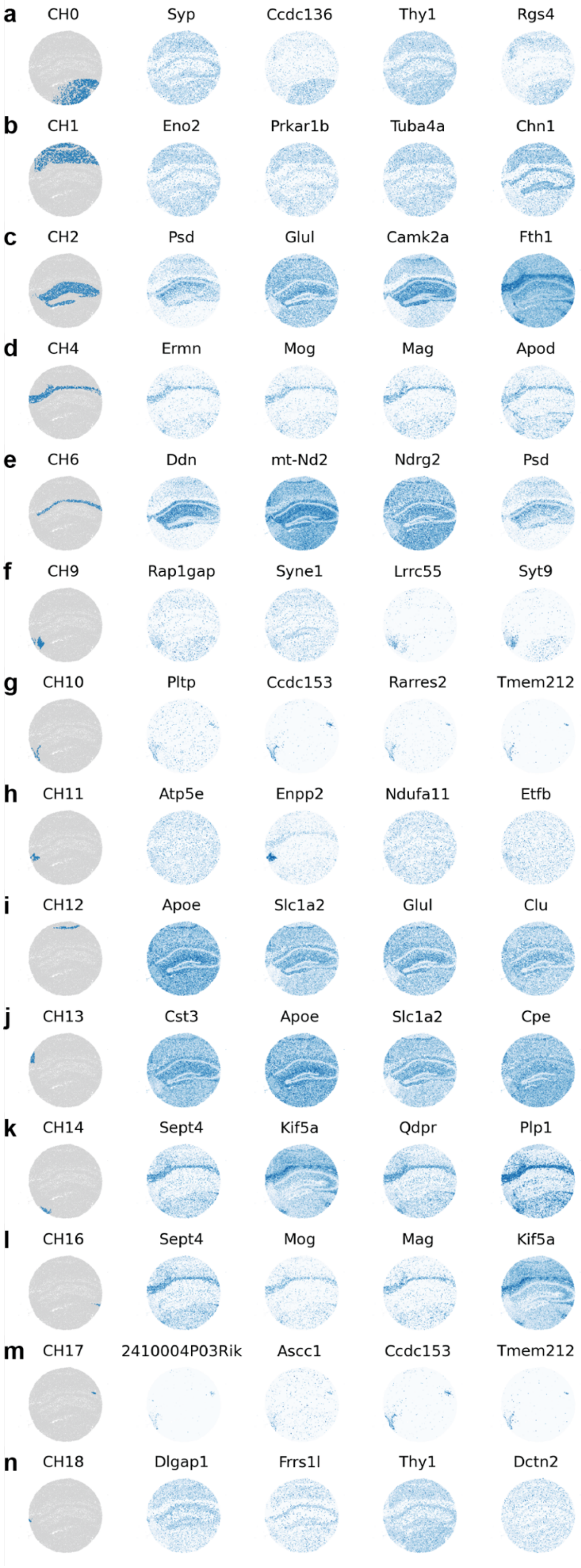
a
CH0
Syp
Ccdc136
Thy1
Rgs4
b
CH1
Eno2
Prkar1b
Tuba4a
Chn1
c
CH2
Psd
Glul
Camk2a
Fth1
d
CH4
Ermn
Mog
Mag
Apod
e
CH6
Ddn
mt-Nd2
Ndrg2
Psd
f
CH9
Rap1gap
Syne1
Lrrc55
Syt9
g
CH10
Pltp
Ccdc153
Rarres2
Tmem212
h
CH11
Atp5e
Enpp2
Ndufa11
Etfb
i
CH12
Apoe
Slc1a2
Glul
Clu
j
CH13
Cst3
Apoe
Slc1a2
Cpe
k
CH14
Sept4
Kif5a
Qdpr
Plp1
l
CH16
Sept4
Mog
Mag
Kif5a
m
CH17
2410004P03Rik
Ascc1
Ccdc153
Tmem212
n
CH18
Dlgap1
Frrs1l
Thy1
Dctn2

**Supplemental Figure 2: a-n** Examples of constituent genes that make up each of the coexpression hotspots outside of the hippocampal structure (all except CH3, CH5, CH7, CH8, and CH15), as well as their associated coexpression hotspot. Note: genes displayed are not sorted by differential expression or other measure. Left column: spots inside the coexpression hotspots are highlighted in blue. Remaining columns: color represents normalized gene expression.

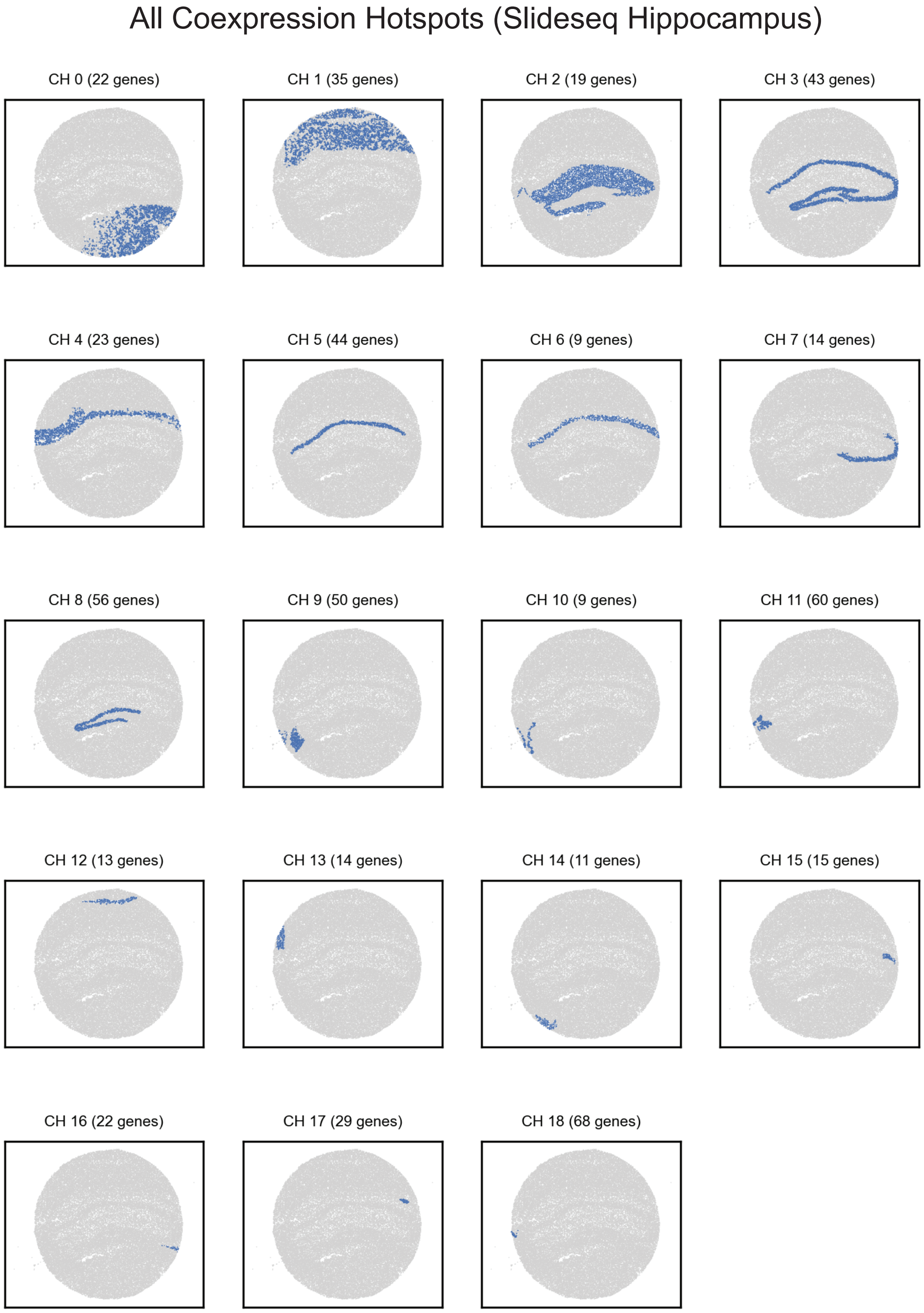


**Supplemental Figure 3:** Individual spatial visualizations of all coexpression hotspots identified in the hippocampus dataset. For comparison, CH 3, 5, 15, 7, and 8 represent the full hippocampus, CA1, CA2, CA3, and dentate gyrus regions, respectively. Spots inside the coexpression hotspots are highlighted in blue.

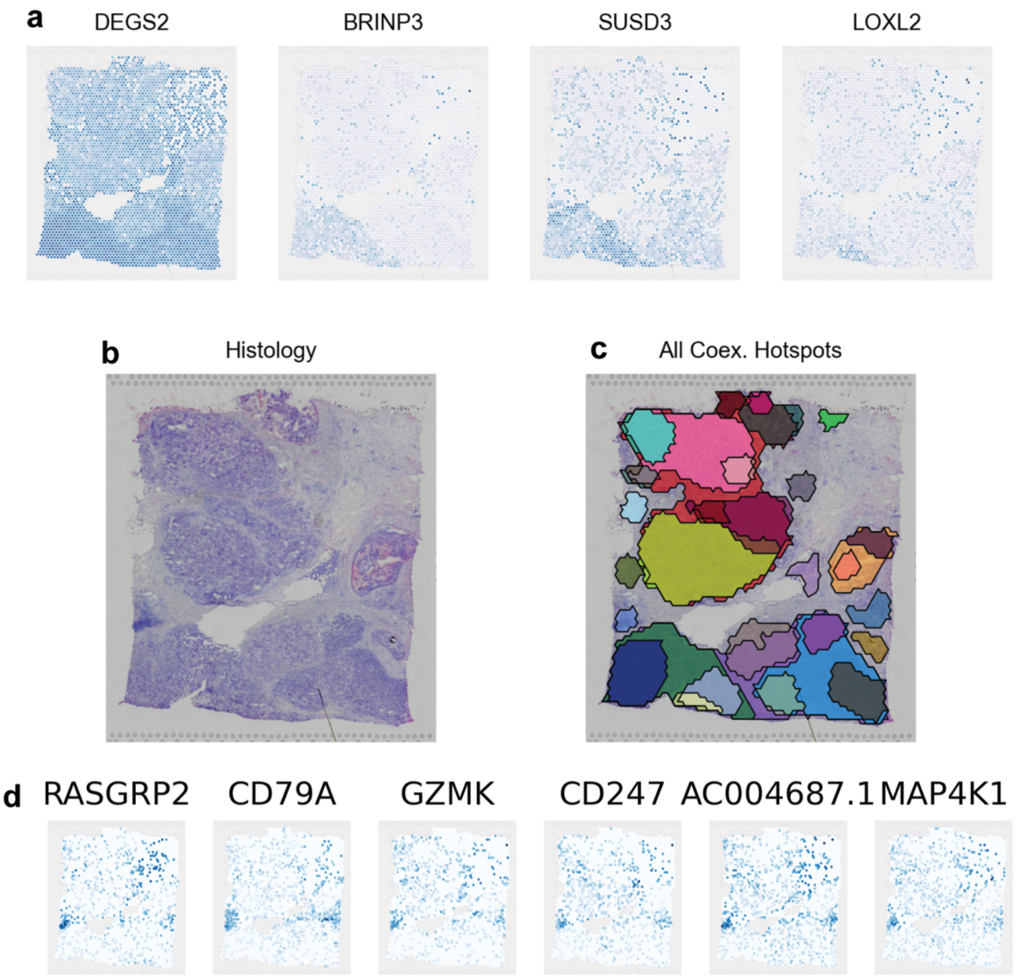


**Supplemental Figure 4: a** Marker genes for the four layers of structure shown in Fig. 3b. Color represents normalized gene expression. **b** Histology image for this dataset (compare to all coexpression hotspots in **c** or the spatial coherence in Fig. 3e). **d** Top-6 marker genes for the TLS region identified by NeST, showing concentrated expression in the TLS area (Fig. 3f). Color represents normalized gene expression.

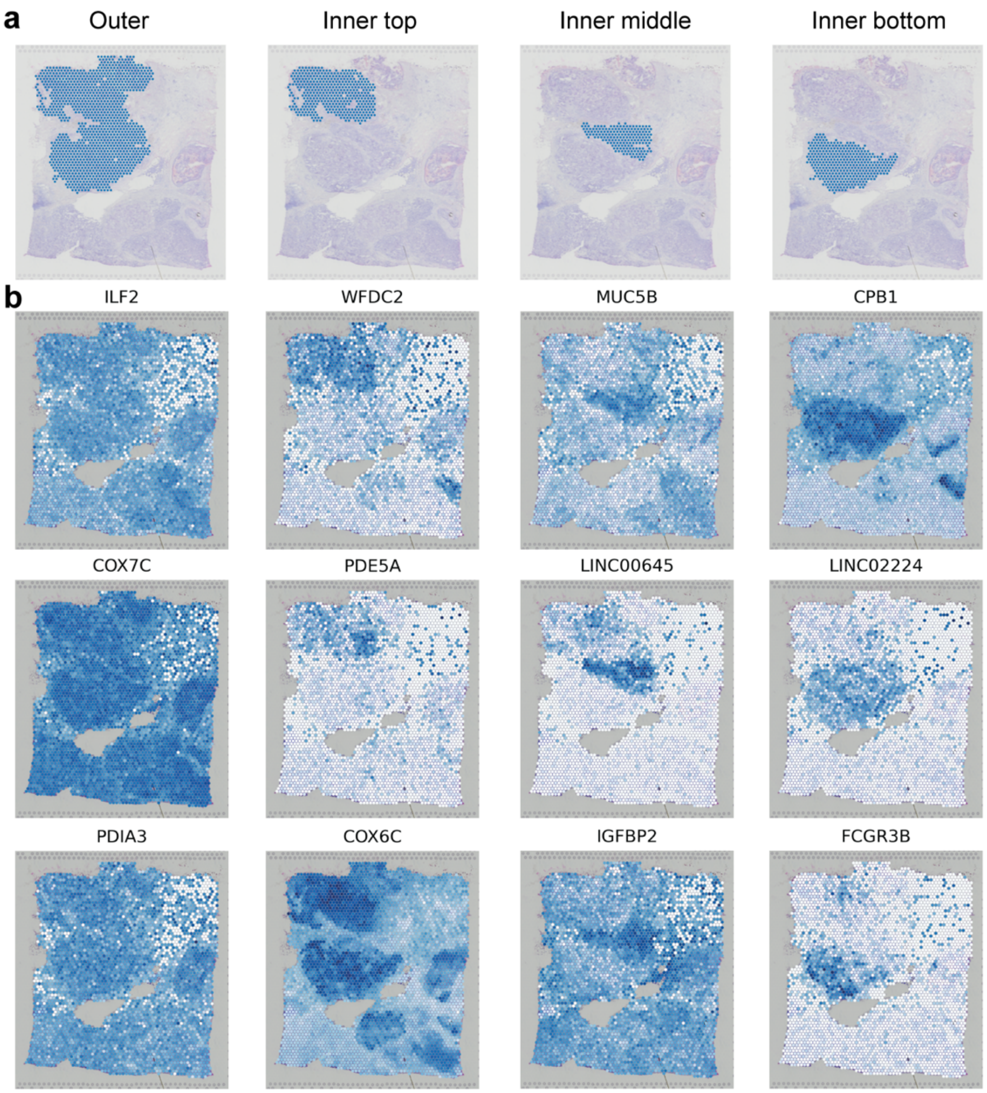


**Supplemental Figure 5: Breast cancer dataset benchmarking regions. a** Hierarchical tumor regions used in the comparison shown in Fig. 4ab. Spots within the coexpression hotspot are highlighted in blue. **b** Examples of specific genes that are localized to each of the four regions shown in **a**. Color represents normalized gene expression.

a

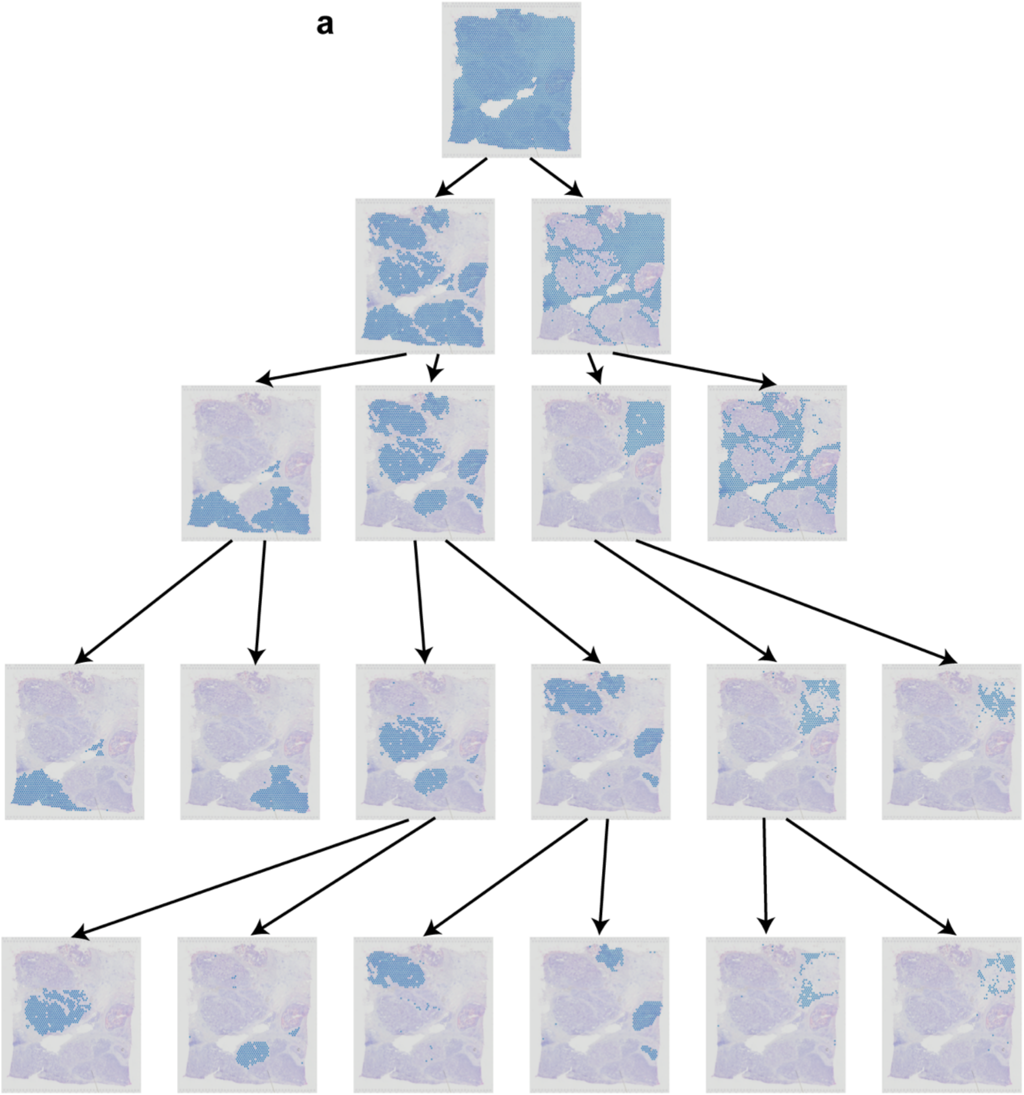

b

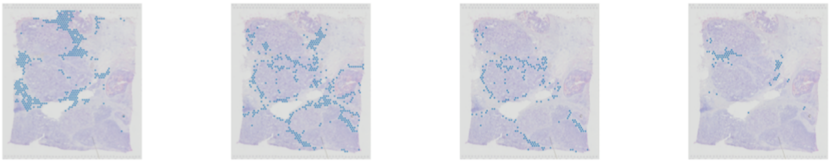

**Supplemental Figure 6. a** A subset of the top of the hierarchical tree computed using hierarchical agglomerative clustering (Ward linkage) on the breast cancer Visium dataset. Note that the tree, many clusters are not meaningfully spatially localized, or are subdivided in a spatially inconsistent manner. Thus use of such hierarchical agglomerative clustering would still require a separate procedure to extract the actual nested hierarchical structure. Spots within each cluster are highlighted in blue. Horizontally aligned panels are in the same level of the hierarchical tree. **b** Other examples of other groups present in the tree that are not clearly spatially localized.

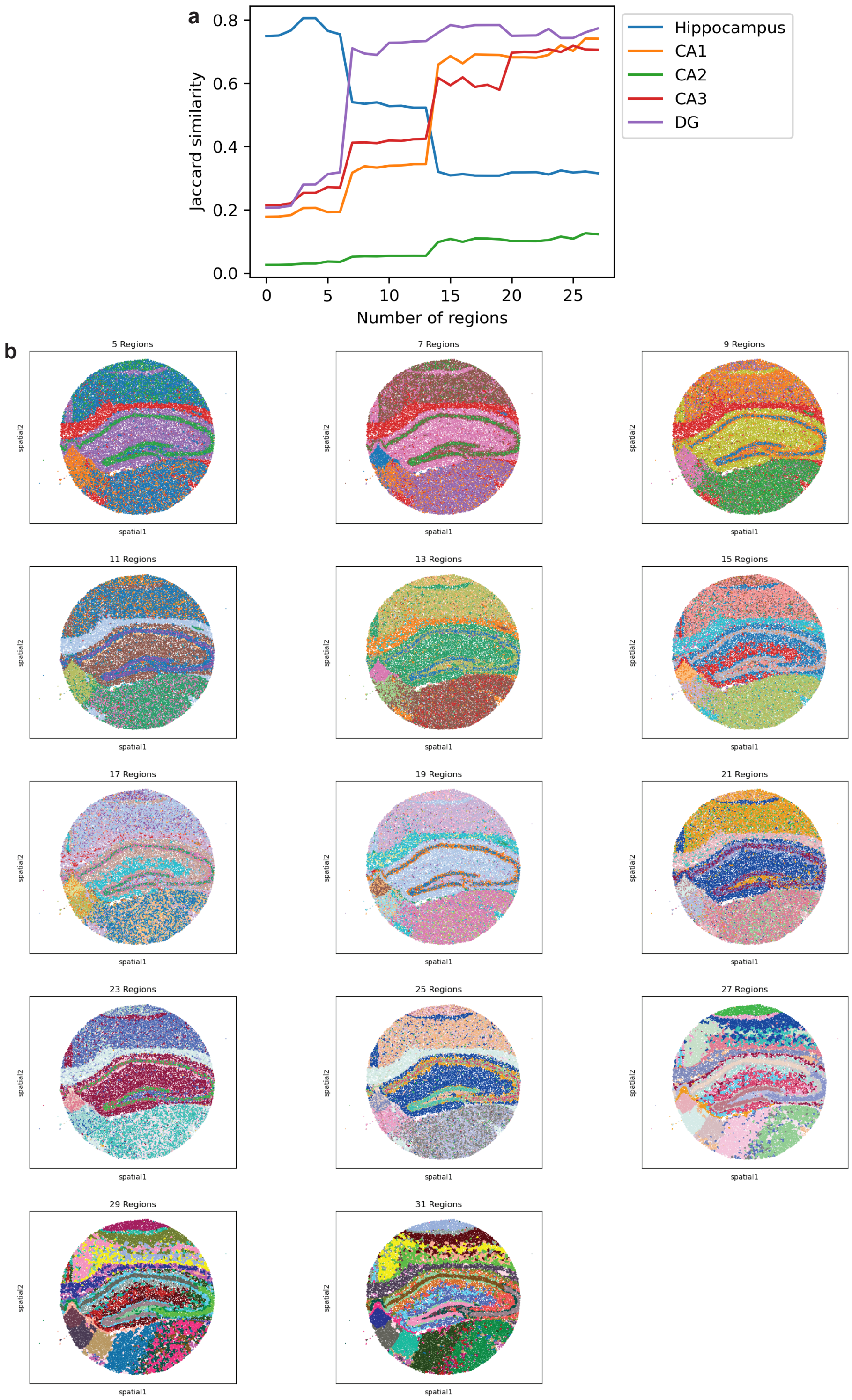


**Supplemental Figure 7. a** Jaccard similarity between NeST coexpression hotspots representing the hippocampus and HMRF regions. At approximately 12 regions, HMRF switches from detecting the full hippocampus structure (coexpression hotspot CH3) to detecting the CA1, CA3, and DG (dentate gyrus) regions, but is not able to find the smaller CA2 region. **b** Region segmentations identified by HMRF for number of regions varying from 5 to 31. Contrast for example n=27, where the CA1, CA3, and DG are clearly separated, with n=9, where they are not. Source data are provided as a Source Data file.

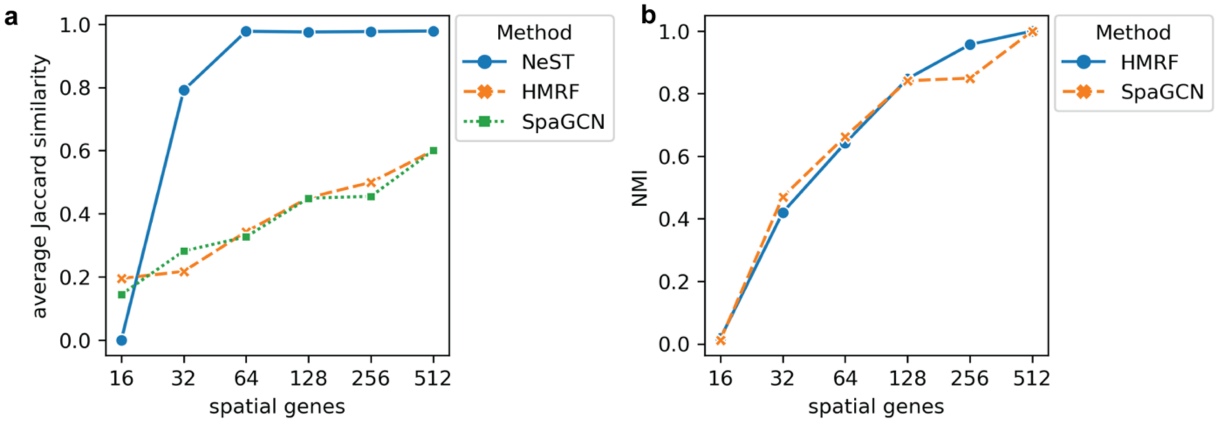


**Supplemental Figure 8: Performance of segmentation methods on synthetic dataset.** **a** Computing the average matched Jaccard similarity shows NeST captures much of the structure with only 32 genes and fully captures the structure with 64 genes. Segmentation methods have lower performance, in part due to their inability to represent hierarchical structures. **b** Comparing the NMI between segmentation and a non-hierarchical representation of the true regions (i.e. a spot in regions 1 through 3 is just labeled 3, a spot in regions 1 through 5 is just labeled 5) shows that even without the hierarchical element the segmentation methods require a much higher number of spatial genes to fully capture the structure compared to NeST. Source data are provided as a Source Data file.

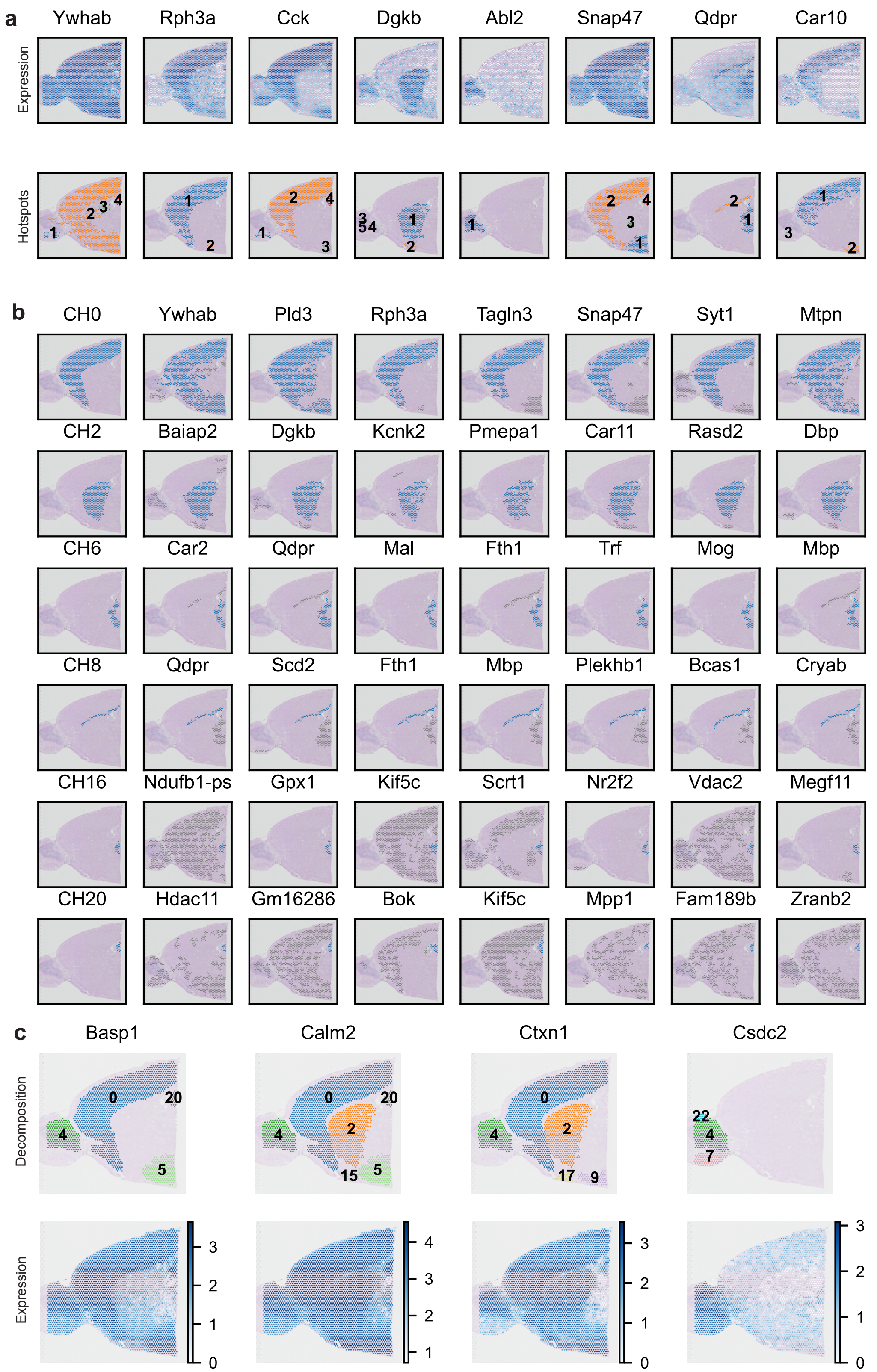


**Supplemental Figure 9: a** Examples of genes exhibiting different expression patterns and their corresponding hotspots. **b** Constituent genes for each of the hotspots considered in Fig. 4a, showing the explainability of coexpression hotspots. **c** Examples of the hotspot decomposition feature of NeST, in which

coexpression hotspots are used to make a best approximation for the overall expression of each gene. Color represents normalized gene expression.

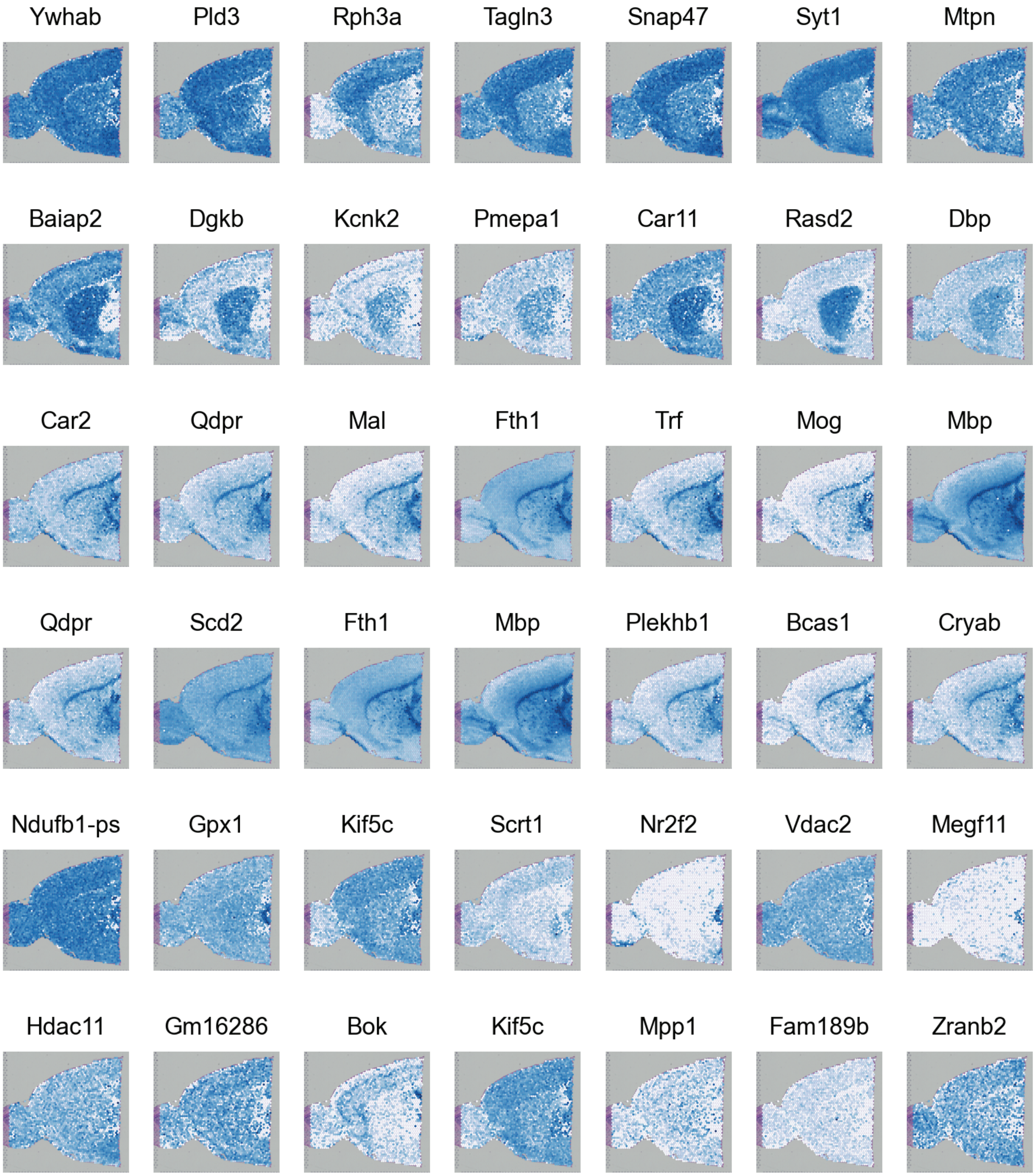


**Supplemental Figure 10:** Underlying gene expression for each of the genes shown in SI Fig. 9. Note that only spots with high expression (relative to the overall expression of that particular gene) and surrounded by other high-expression spots are actually included in hotspots and seen in SI Fig. 9. Color represents normalized gene expression.

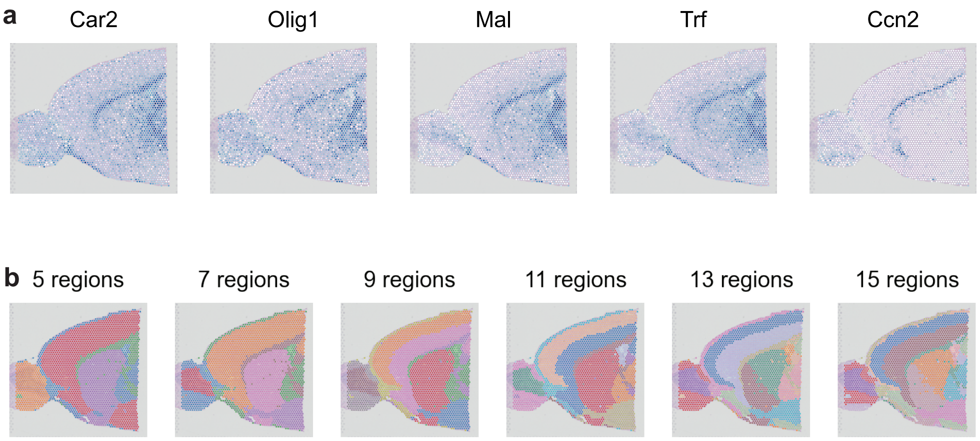


**Supplemental Figure 11: a** Examples from the second slice of the mouse anterior cortex Visium dataset, showing the same pattern in *Ccn2* behavior as seen in the first slice. Color represents normalized gene expression. **b** HMRF segmentations of the dataset for various numbers of regions, showing the difficulty in correctly identifying the CH6 and CH8 as distinct areas. Color indicates different HMRF regions.

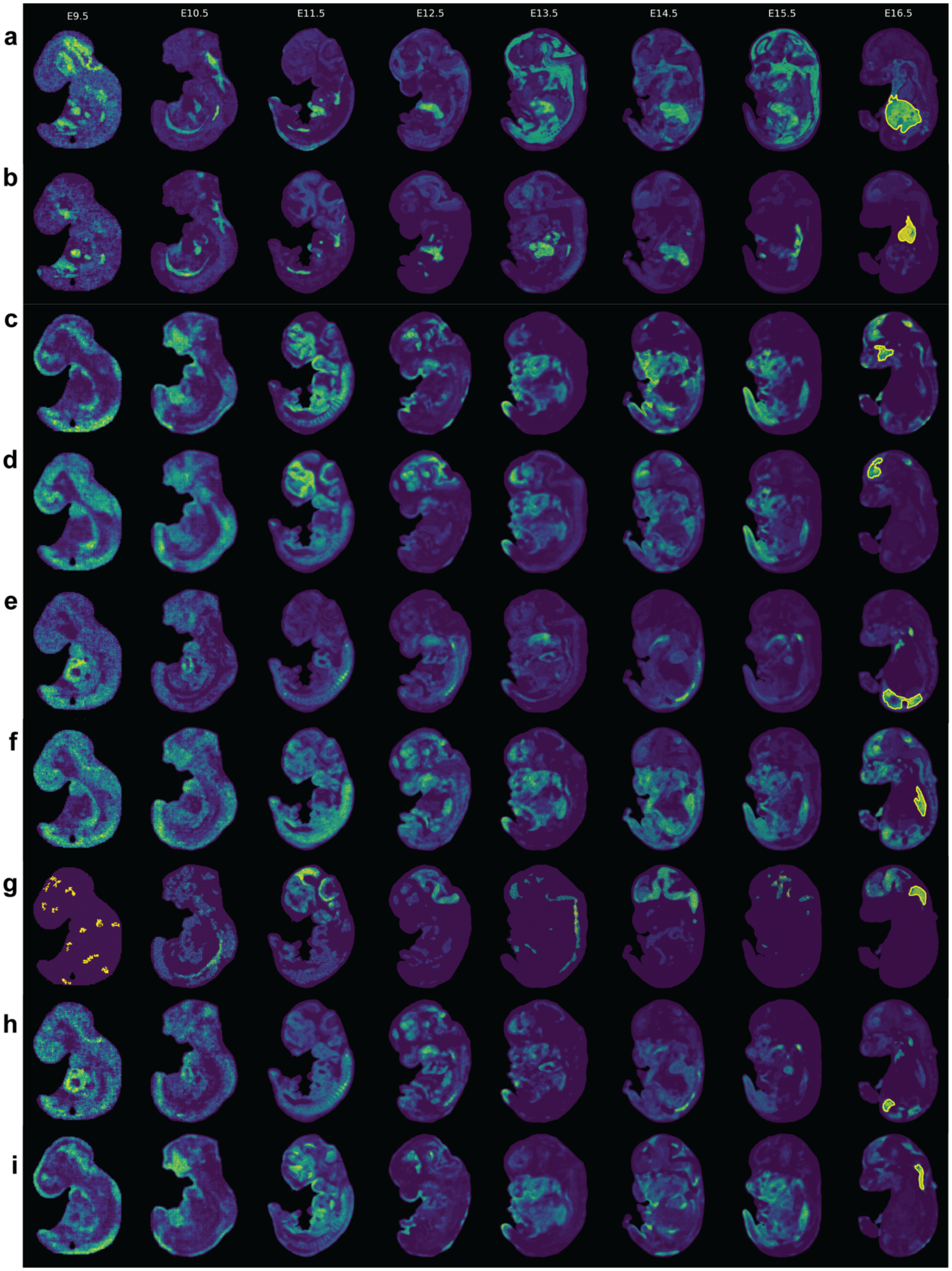


**Supplemental Figure 12: a-i** Similarity maps comparing coexpression hotspots CH1-CH9 identified in the final E16.5 sample to all previous samples, showing where in the previous samples similar genes were spatially expressed (also see Fig. 5g). Color represents normalized gene expression.

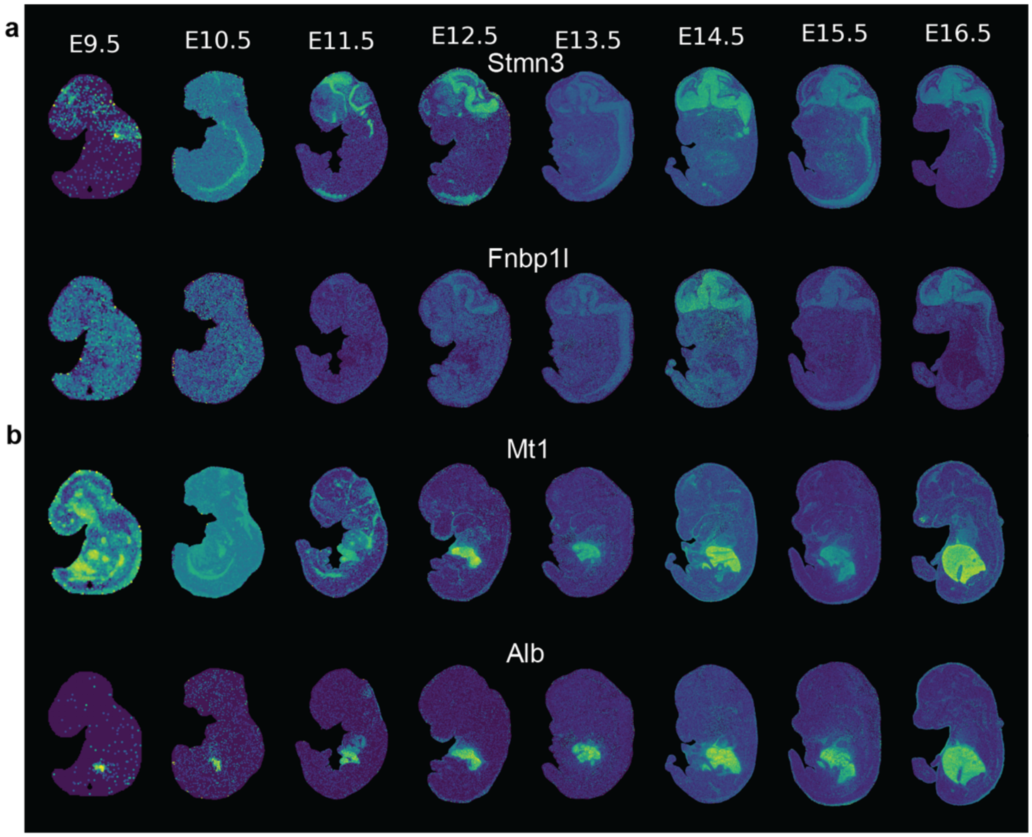


**Supplemental Figure 13:** Evolution of expression of genes associated with the (**a)** E16.5 CH0 (brain), shown in Fig. 5b, and (**b)** CH1 (liver), shown in SI Fig. 12a. Color represents normalized gene expression.

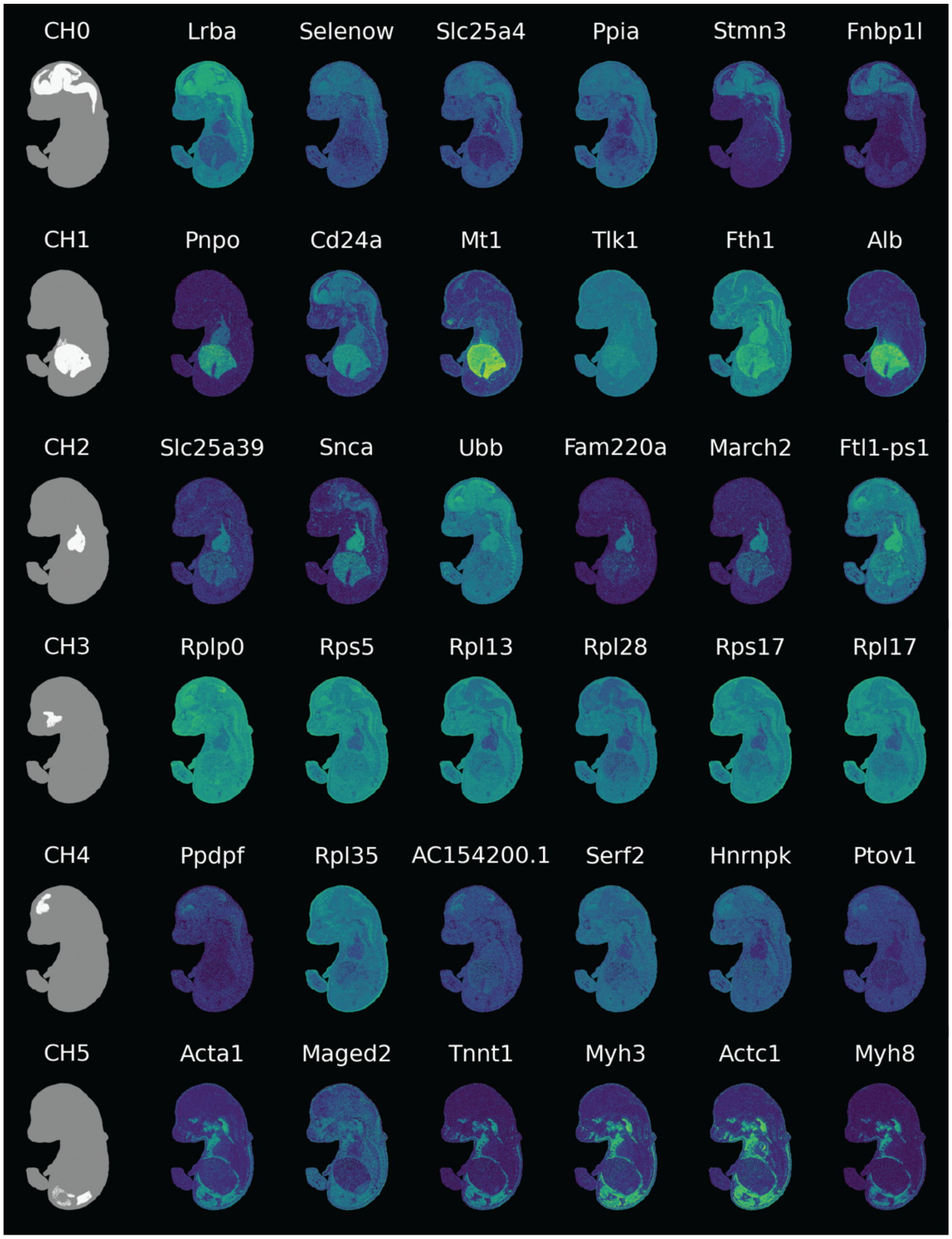


**Supplemental Figure 14:** 6 constituent genes each shown for each CH0 through CH5 (CH0 shown in Fig. 5g, CH1-5 shown in SI Fig. 12a-e). Color represents normalized gene expression, and the location of the coexpression hotspot highlighted in white.

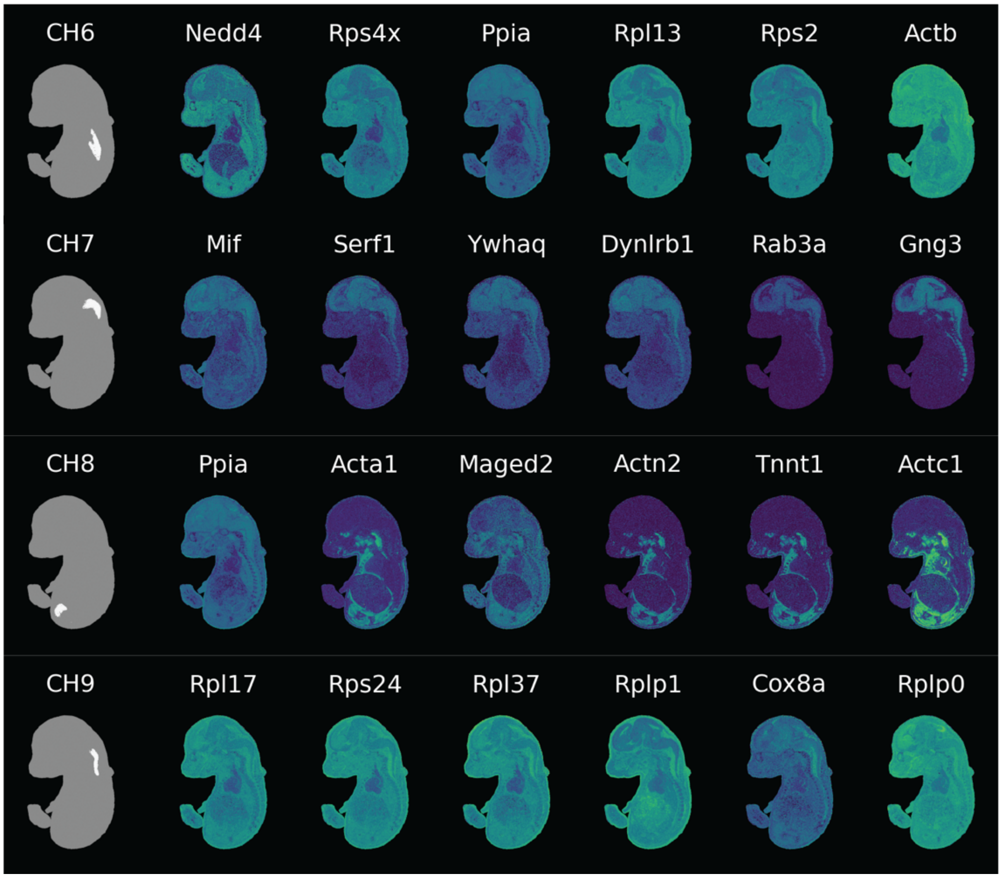


**Supplemental Figure 15:** 6 constituent genes each shown for each CH6 through CH9 (shown in SI Fig. 12f-i). Color represents normalized gene expression, and the location of the coexpression hotspot highlighted in white.

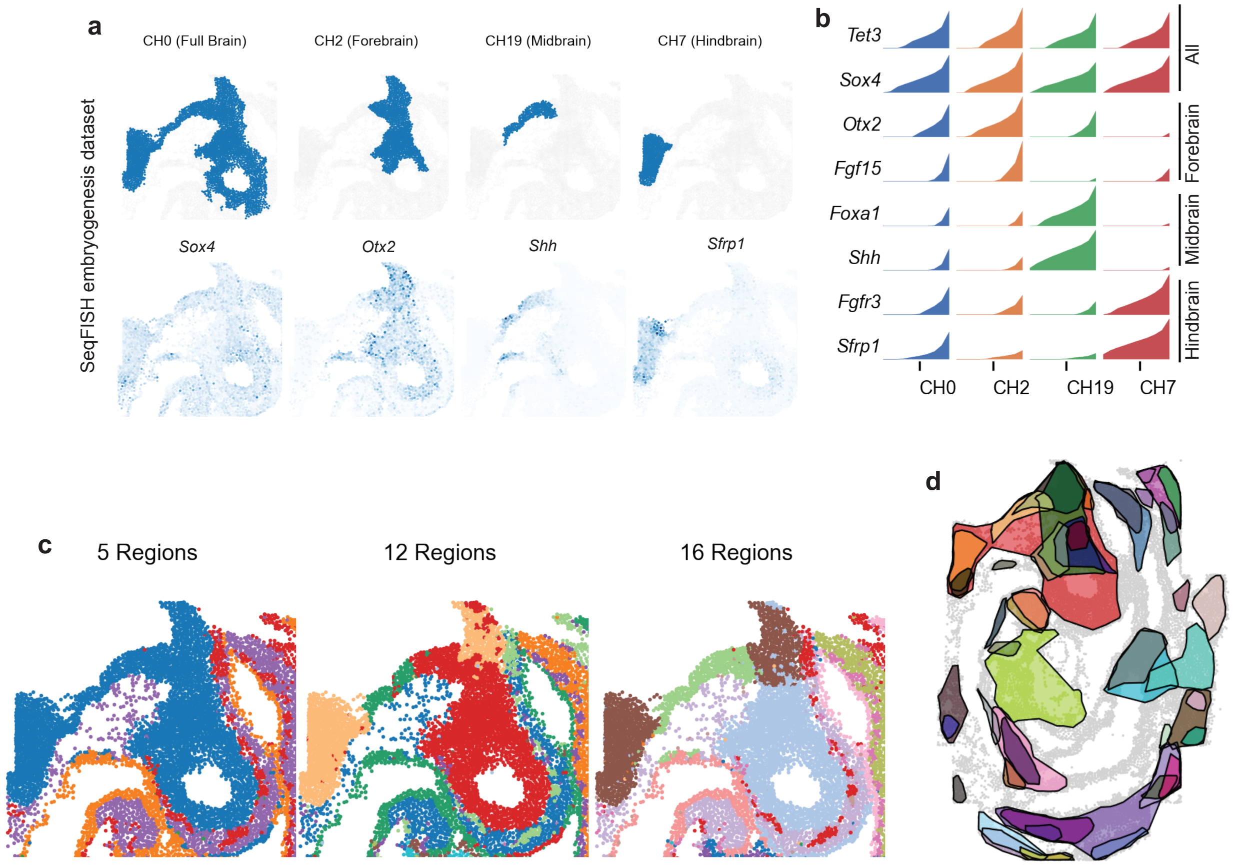


**Supplemental Figure 16: a** Coexpression hotspots and example constituent genes showing the nested structure in the brain area, with hotspots representing the full brain, as well as the forebrain, midbrain, and hindbrain areas. Top row: cells within each coexpression hotspot are highlighted in blue. Bottom row: color represents normalized gene expression. **b** Tracks plot of top-2 marker genes for each area. **c** HMRF segmentations for three different numbers of regions, showing that even though the brain is subdivided, the divisions do not line up with the forebrain/midbrain/hindbrain structure. Color identifies different regions. **d** All coexpression hotspots for this dataset. Color identifies different coexpression hotspots.

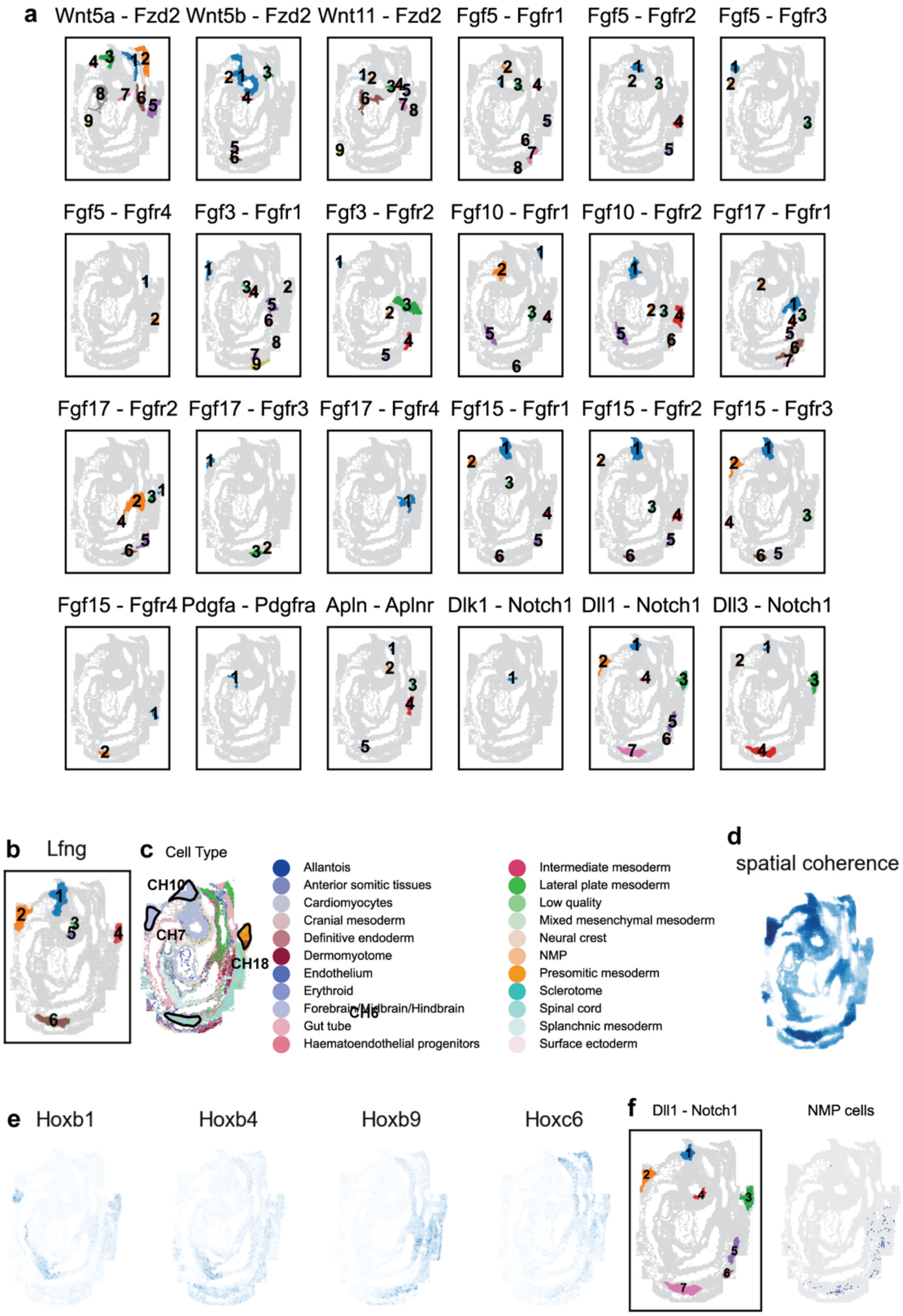


**Supplemental Figure 17: a** CCI hotspots identified by NeST for each of the 24 identified L-R interactions. Note similarity between *Notch1* and *Fgf15* interactions. **b** Hotspots for the gene *Lfng* which is known to be associated with Notch signaling, showing expression in the same areas as are active in Notch signaling. **c** Cell type annotation for the dataset overlaid with the four Notch-active hotspots, showing that all except CH18 have high cell type heterogeneity within the coexpression hotspot. **d** Spatial coherence score computed by NeST. **e** Spatial expression of *Hox* genes that were highly differentially expressed in Fig. 5g. **f** Comparison of *Dll1-Notch1* hotspots to NMP cells, showing that despite the high overlap fraction there is not a clear spatial agreement between the two.

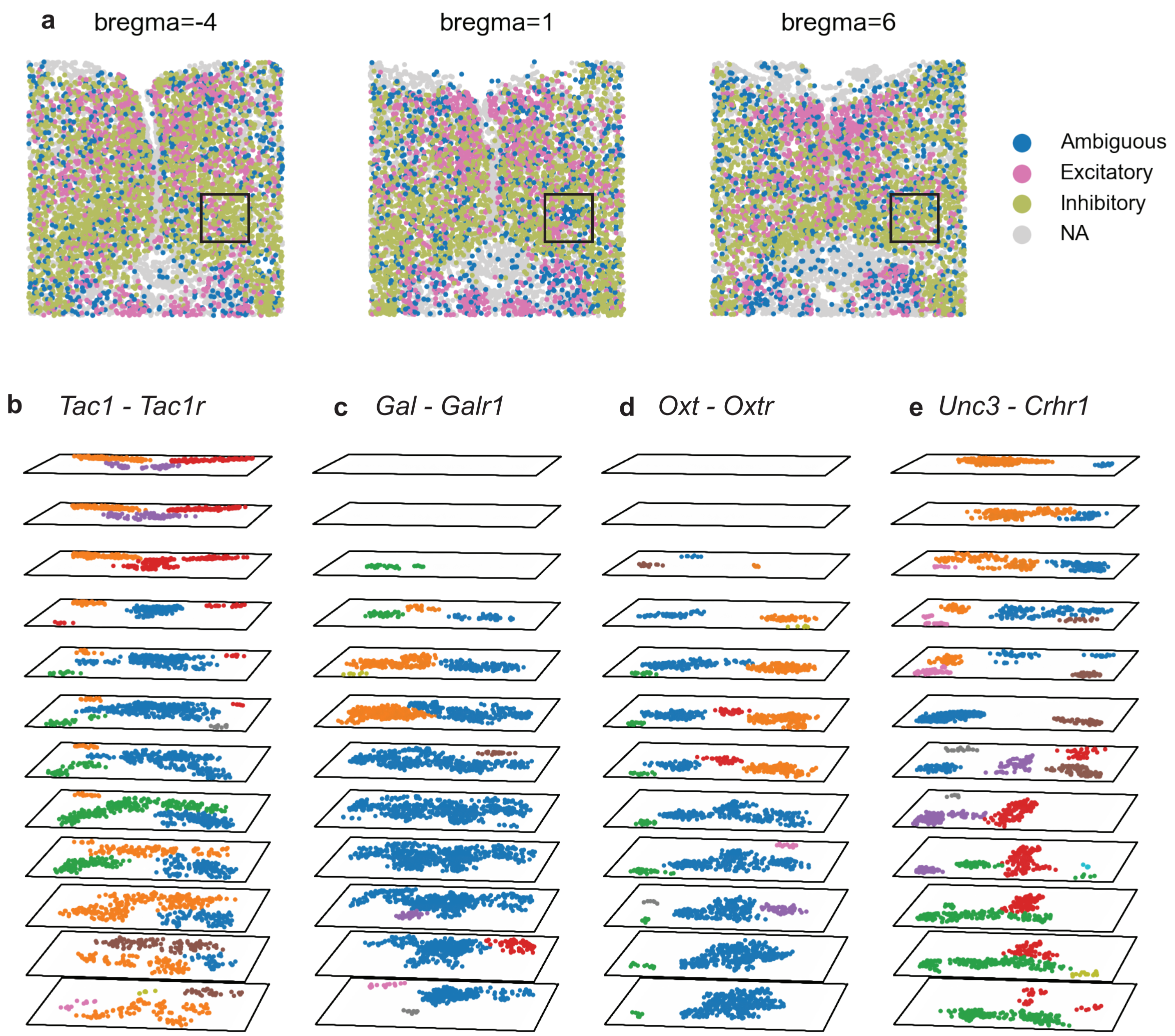


**Supplemental Figure 18: a** Cell type annotations *Ambiguous, Excitatory,* and *Inhibitory* are the primary types associated in the signaling event shown in Fig. 6b, but the spatial positions of these cell types is not localized to the same area (area from Fig. 6b shown with overlaid box). **b-e** Examples of 3D CCI hotspots identified with NeST for four different L-R interactions. *Tac1 – Tac1r* (leftmost) are investigated further in Fig. 6f-n. Color identifies distinct 3D hotspots. Labeling is shared across all layers.

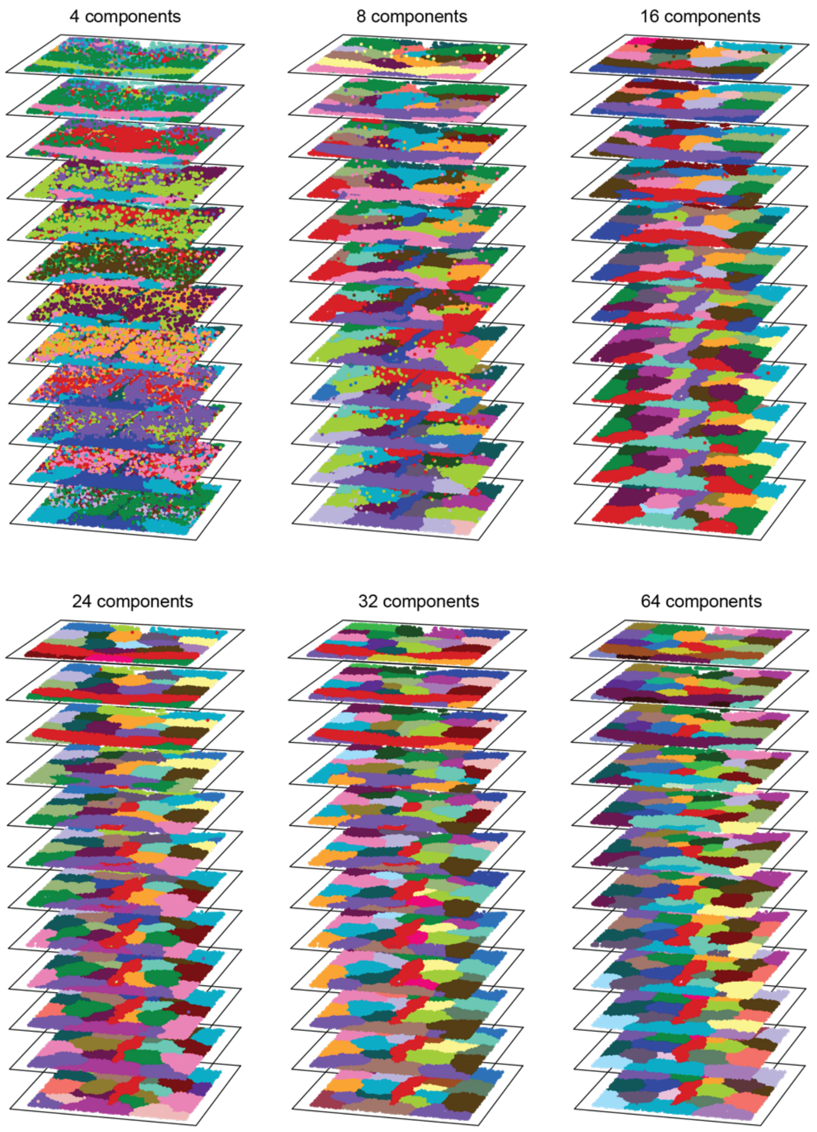


**Supplemental Figure 19.** 3D region visualizations computing using varying numbers of principal components in the PCA decomposition of the expression (c.f. Fig. 7e). Coloring identifies distinct 3D regions.
