# Supplementary material for: NeST: nested hierarchical structure identification in spatial transcriptomic data
Source: Nat Commun. 2023 Oct 17;14:6554. doi: 10.1038/s41467-023-42343-x (PMC10582109; doi:10.1038/s41467-023-42343-x)
Supplement: Supplementary file 3 — Reporting Summary [file 41467_2023_42343_MOESM3_ESM.pdf]

nature portfolio

Corresponding author(s): Qing Nie

Last updated by author(s): Sep 11, 2023

Reporting Summary

Nature Portfolio wishes to improve the reproducibility of the work that we publish. This form provides structure for consistency and transparency in reporting. For further information on Nature Portfolio policies, see our Editorial Policies and the Editorial Policy Checklist.

Statistics

For all statistical analyses, confirm that the following items are present in the figure legend, table legend, main text, or Methods section.

n/a	Confirmed	
☐	☒	The exact sample size (*n*) for each experimental group/condition, given as a discrete number and unit of measurement
☐	☒	A statement on whether measurements were taken from distinct samples or whether the same sample was measured repeatedly
☐	☒	The statistical test(s) used AND whether they are one- or two-sided<br>*Only common tests should be described solely by name; describe more complex techniques in the Methods section.*
☐	☒	A description of all covariates tested
☐	☒	A description of any assumptions or corrections, such as tests of normality and adjustment for multiple comparisons
☐	☒	A full description of the statistical parameters including central tendency (e.g. means) or other basic estimates (e.g. regression coefficient) AND variation (e.g. standard deviation) or associated estimates of uncertainty (e.g. confidence intervals)
☐	☒	For null hypothesis testing, the test statistic (e.g. *F*, *t*, *r*) with confidence intervals, effect sizes, degrees of freedom and *P* value noted<br>*Give P values as exact values whenever suitable.*
☒	☐	For Bayesian analysis, information on the choice of priors and Markov chain Monte Carlo settings
☒	☐	For hierarchical and complex designs, identification of the appropriate level for tests and full reporting of outcomes
☒	☐	Estimates of effect sizes (e.g. Cohen's *d*, Pearson's *r*), indicating how they were calculated

Our web collection on statistics for biologists contains articles on many of the points above.

Software and code

Policy information about availability of computer code

Data collection	No experimental data was collected in this study.
Data analysis	Data analysis was performed in Python using our method NeST, which is made publicly available at https://github.com/bwalker1/NeST. The official release of NeST along with this manuscript will be labeled version 1.0 and tagged with Zenodo. Principal requirements for NeST are Python>=3.9, R>=4.2, Cellchat=1.5.0, Scanpy=1.8.2, Squidpy=1.2. Comparison was performed against BayesSpace=1.5.1, SpaGCN=1.2, and a custom implementation of HMRF included in NeST. A complete list of all packages used in a NeST Python environment (which numbers in the hundreds), as well as a Docker image for exact reproduction of a standardized NeST environment, are accessible on the NeST Github.

For manuscripts utilizing custom algorithms or software that are central to the research but not yet described in published literature, software must be made available to editors and reviewers. We strongly encourage code deposition in a community repository (e.g. GitHub). See the Nature Portfolio guidelines for submitting code & software for further information.

Data

Policy information about availability of data

All manuscripts must include a data availability statement. This statement should provide the following information, where applicable:

- Accession codes, unique identifiers, or web links for publicly available datasets
- A description of any restrictions on data availability
- For clinical datasets or third party data, please ensure that the statement adheres to our policy

Visium 10x were accessed via SCANPY54. Other datasets were used through the Squidpy package61. Necessary code to load all datasets and use them with NeST is

available as part of the NeST package. Raw forms of transcriptomic datasets are also available from the original authors. The Visium 10x datasets used in this study are available in 10x Genomics database at https://support.10xgenomics.com/spatial-gene-expression/datasets. The Slide-seqV2 dataset used in this study is available in the Single Cell Portal database at https://singlecell.broadinstitute.org/single_cell/study/SCP815/highly-sensitive-spatial-transcriptomics-at-near-cellular-resolution-with-slide-seqv2. The SeqFISH dataset used in this study is available in the Spatial Mouse Atlas database at https://marionilab.cruk.cam.ac.uk/SpatialMouseAtlas/. The MERFISH dataset used in this study is available in Dryad at https://doi.org/10.5061/dryad.8t8s248. The intestine colitis Visium dataset used in this study is available in the GEO database at https://www.ncbi.nlm.nih.gov/geo/query/acc.cgi?acc=GSE169749. The Stereo-seq data of mouse embryo development is available in the STOMICS database at https://db.cngb.org/stomics/mosta/download/. The CellChat database of ligand-receptor interactions used in this study is part of the CellChat R library available at https://github.com/sqjin/CellChat.

Research involving human participants, their data, or biological material

Policy information about studies with human participants or human data. See also policy information about sex, gender (identity/presentation), and sexual orientation and race, ethnicity and racism.

Reporting on sex and gender	This study does not involve human research participants.
Reporting on race, ethnicity, or other socially relevant groupings	This study does not involve human research participants.
Population characteristics	This study does not involve human research participants.
Recruitment	This study does not involve human research participants.
Ethics oversight	This study does not involve human research participants.

Note that full information on the approval of the study protocol must also be provided in the manuscript.

Field-specific reporting

Please select the one below that is the best fit for your research. If you are not sure, read the appropriate sections before making your selection.

[x] Life sciences [ ] Behavioural & social sciences [ ] Ecological, evolutionary & environmental sciences

For a reference copy of the document with all sections, see nature.com/documents/nr-reporting-summary-flat.pdf

Life sciences study design

All studies must disclose on these points even when the disclosure is negative.

Sample size	Analysis was performed on a total of seven datasets, plus one synthetic dataset. These datasets comprehensively cover the range of variation in spatial transcriptomic datasets, in both spatial resolution and gene coverage, present in the literature, and thus are sufficient to demonstrate the validity of the method in a wide range of conditions.
Data exclusions	When NeST was applied to a dataset, it was applied over all genes and all cells/spots/spatial points. For the MOSTA Stereo-seq dataset, the intent was to visualize temporal change and so one sample was chosen for each of the eight time points (From E9.5 to E16.5). In each case the sample labeled Embryo 1 Sample 1 (E1S1) was chosen, which we considered the most natural choice, and this choice was made prior to any analysis being performed.
Replication	NeST is a deterministic computational method, and is necessarily guaranteed to produce the same results when applied to the same input data. Our choice of datasets and analyses covers a wide range of possible spatial transcriptomic technologies, reinforcing the reproducibility of the conclusions.
Randomization	There was no allocation of samples into experimental groups. This study used a collection of previously published, publically available datasets, and computational analysis of each dataset was performed independently.
Blinding	Investigators were not blinded to any aspect of the study. No data was gathered or organized into experimental groups, and there were no aspects of the study in which it would make sense to perform blinding, or in which it is previously established in the literature to perform blinding.

Reporting for specific materials, systems and methods

We require information from authors about some types of materials, experimental systems and methods used in many studies. Here, indicate whether each material, system or method listed is relevant to your study. If you are not sure if a list item applies to your research, read the appropriate section before selecting a response.

Materials & experimental systems

n/a	Involved in the study
☒	☐ Antibodies
☒	☐ Eukaryotic cell lines
☒	☐ Palaeontology and archaeology
☒	☐ Animals and other organisms
☒	☐ Clinical data
☒	☐ Dual use research of concern
☒	☐ Plants

Methods

n/a	Involved in the study
☒	☐ ChIP-seq
☒	☐ Flow cytometry
☒	☐ MRI-based neuroimaging
